# NEMO regulates a cell death switch in TNF signaling by inhibiting recruitment of RIPK3 to the cell death-inducing complex II

**DOI:** 10.1038/cddis.2016.245

**Published:** 2016-08-25

**Authors:** Alessandra Pescatore, Elio Esposito, Peter Draber, Henning Walczak, Matilde Valeria Ursini

**Affiliations:** 1 Institute of Genetics and Biophysics ‘Adriano Buzzati-Traverso' (CNR), Naples 80131, Italy; 2 Centre for Cell Death, Cancer, and Inflammation (CCCI), UCL Cancer Institute, University College London, 72 Huntley Street, London WC1E 6BT, UK

## Abstract

Incontinentia Pigmenti (IP) is a rare X-linked disease characterized by early male lethality and multiple abnormalities in heterozygous females. IP is caused by NF-*κ*B essential modulator (NEMO) mutations. The current mechanistic model suggests that NEMO functions as a crucial component mediating the recruitment of the I*κ*B-kinase (IKK) complex to tumor necrosis factor receptor 1 (TNF-R1), thus allowing activation of the pro-survival NF-*κ*B response. However, recent studies have suggested that gene activation and cell death inhibition are two independent activities of NEMO. Here we describe that cells expressing the IP-associated NEMO-A323P mutant had completely abrogated TNF-induced NF-*κ*B activation, but retained partial antiapoptotic activity and exhibited high sensitivity to death by necroptosis. We found that robust caspase activation in NEMO-deficient cells is concomitant with RIPK3 recruitment to the apoptosis-mediating complex. In contrast, cells expressing the ubiquitin-binding mutant NEMO-A323P did not recruit RIPK3 to complex II, an event that prevented caspase activation. Hence NEMO, independently from NF-*κ*B activation, represents *per se* a key component in the structural and functional dynamics of the different TNF-R1-induced complexes. Alteration of this process may result in differing cellular outcomes and, consequently, also pathological effects in IP patients with different NEMO mutations.

Tumor necrosis factor *α* (TNF) is a major cytokine promoting inflammation and innate immune responses. Depending on the cell type, exposure to TNF can result in either cell survival or death, reflecting an intricate network of signals that are triggered by this molecule upon binding to its cognate receptors of which TNF-receptor 1 (TNF-R1) is the major one.^1^

Following ligand binding, TNF-R1 undergoes trimerization and a conformational change that triggers the recruitment of multiple adaptors, including ubiquitin ligases and kinases, leading to the formation of the receptor-associated complex I.^1^ This includes, besides the receptor and the crosslinking ligand, the adaptor proteins TRADD and TRAF2, the kinase RIPK1, and the E3 ubiquitin ligases cellular inhibitor of apoptosis protein (cIAP) 1 and cIAP2. cIAP1/2 mediate the ubiquitination of several components of the complex I, leading to recruitment of both the TAK1-TAB1-TAB2 and the linear ubiquitin chain assembly complex (LUBAC). LUBAC in turn mediates the formation of linear ubiquitin chains which enhances recruitment of the I*κ*B-kinase (IKK) complex, composed of the adaptor protein NEMO (NF-*κ*B essential modulator) and the kinases IKK1 and IKK2.^2^ NEMO specifically binds to non-degradative ubiquitin chains within the TNF-R1 signaling complex through the cooperation of two domains: the NEMO ubiquitin-binding domain (NUB, also called UBAN) and the zinc-finger domain. Together, they enable efficient recruitment and retention of IKK1/2 and their activation by the TAK1.^3, 4, 5^ The IKK complex subsequently activates NF-*κ*B signaling, leading to transcription of pro-survival genes, such as cIAP1/2, and the cellular FLICE-like inhibitor protein (cFLIP), an enzyme-inactive homolog of Caspase-8.^6, 7, 8^

Following internalization of TNF-R1, a cytoplasmic complex II consisting of RIPK1, FADD (Fas-Associated protein with Death Domain) and the Caspase-8 is formed.^9^ Normally, apoptosis is prevented by dimerization between Caspase-8 and cFLIP. If the cFLIP levels are low, such as in the absence of NEMO, active dimers of Caspase-8 are formed driving apoptosis.^10^ Active Caspase-8 not only initiates the apoptotic program but also cleaves and inactivates essential necroptosis mediators such as RIPK1, RIPK3 and CYLD.^11, 12, 13^

When Caspase-8 activity is compromised, the RIPK1–RIPK3 oligomerization leads to RIPK3 autoactivation that in turn phosphorylates MLKL.^14, 15, 16, 17^ MLKL phosphorylation is the key event required for its translocation to the membrane where it triggers plasma membrane leakage, leading to the execution of ‘necroptotic' cell death.^18^ The interest in necroptosis has grown substantially as several studies highlighted the importance of this form of cell death for various physiological and pathological conditions.^19^

Incontinentia Pigmenti (IP) (OMIM 308300) is a severe X-linked dominant neuro-ectodermal disorder, lethal in males, caused by NEMO mutations. In almost 80% of IP patients, a common deletion occurs, leading to a truncated and faulty NEMO protein. Fibroblasts from male fetuses harboring deleted NEMO show sensitivity to TNF-induced cell death which is thought to be a consequence of the impaired NF-*κ*B-mediated induction of antiapoptotic genes.^20, 21^ Several lines of evidence point towards the possibility that NEMO and IKK1/IKK2 may also be more directly involved in preventing TNF-induced cell death in a manner that is NF-*κ*B-independent.^10, 22, 23^ Few NEMO missense mutations have been reported over the years,^24^ including A323P mutation that was identified in a girl exhibiting the classic IP-skin abnormalities associated with severe neurological defects. This mutation impairs NF-*κ*B activation, even if it was able to interact with IKK catalitic subunits 1 and 2. After the discovery of the NUB domain, it was clearly shown that this mutation abolished NEMO's ability to bind to ubiquitin.^25^

Here, we sought to compare the molecular composition of the TNF-R1-induced signaling complexes and the signaling outcome triggered by TNF in cells completely devoid of NEMO, expressing NEMO-WT or the IP-associated NEMO mutation A323P. We show that NEMO-A323P-expressing cells are unable to induce NF-*κ*B activation. Despite this, these cells partially retain the NEMO antiapoptotic function, yet were markedly sensitized to RIPK3/MLKL-mediated necroptosis. Thereby, we discover a previously unrecognized transcription-independent regulatory function of NEMO in TNF signaling, whose dysregulation contributes to the complexity of diseases associated with mutations in NEMO.

## Results

### Cells expressing NEMO-A323P present abnormalities of cell death induction upon TNF exposure

**Figure 1:**
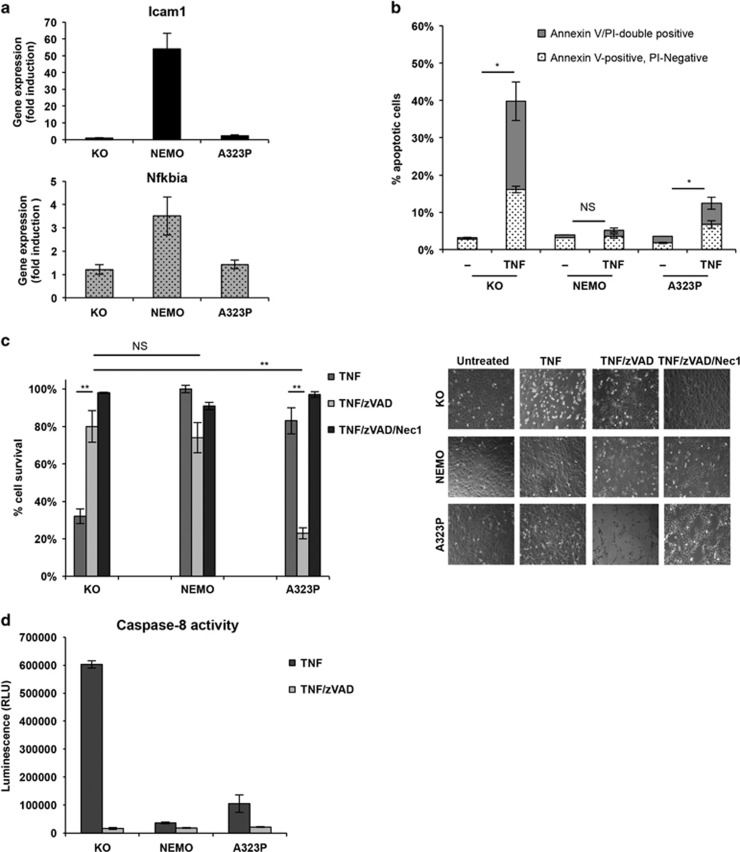
TNF treatment induced different sensitivity to cell death in the NF-*κ*B unresponsive NEMO-KO and NEMO-A323P cells. (**a**) Nemo^(−)^ MEF reconstituted with empty pBabe-puro vector (KO), wild-type NEMO (NEMO) and IP-associated NEMO-A323P mutant (A323P) were treated for 1 h with 10 ng/ml of TNF. Quantitative real-time PCR was performed in triplicate, and the relative abundance of the indicated transcripts (Icam1 and Nfkbia) was calculated with respect to the expression of Hprt transcript. Error bars represent S.D. of triplicates experiment. (**b**) KO, NEMO and A323P expressing MEF cells treated with TNF for 6 h. Apoptosis was measured after double staining with Annexin-V and PI. Percentage of Annexin-V/PI double-positive cells and Annexin-V positive, PI-negative cells was shown (as indicated). (**c**) KO, NEMO and A323P cells were pre-incubated 1 h with DMSO, zVAD (20 mM) or zVAD in combination with Nec1 (30 μM), followed by 6 h of TNF (20 ng/ml) stimulation. Identical concentrations of these death-inducing agents were used in subsequent experiments unless otherwise stated. Relative cell viability was assessed by determining ATP levels with CellTiter-Glo after 6 h of TNF treatment, as a percentage of untreated cells. Data are presented as mean±S.E.M. (three independent experiments performed), *P-value<0.01; **P-value<0.001 (Student's *t*-test); NS, not significant. Cells were observed on a phase-contrast microscope (right panel). Representative images of three independent experiments are presented. (**d**) KO, NEMO and NEMO-A323P cells were treated for 2 h with TNF in the presence or absence of zVAD. The luminescence is proportional to Caspase-8 activity, relative light unit (RLU). Error bars represent S.D. of triplicates of one representative experiment of three

In addition to NF-*κ*B activation, TNF is able to induce cell death in a variety of cell lines. NEMO-deficient cells are unable to activate NF-*κ*B upon TNF stimulation and succumb to cell death.^28^ To test whether expression of NEMO-A323P also has functional consequences for TNF-induced cytotoxicity, we reconstituted NEMO-deficient murine embryonic fibroblast (MEF) with empty vector (herein reported as NEMO-KO), wild-type NEMO (NEMO-WT) or NEMO harboring the A323P mutation (NEMO-A323P). Equal expression of NEMO-WT and NEMO-A323P was assessed by real-time PCR (Supplementary Figure S1a). NEMO-KO cell were unable to activate NF-*κ*B upon TNF stimulation and this was rescued by expression of NEMO-WT, but not by expression of the NEMO-A323P mutant (Figure 1a). Accordingly, TNF-induced I*κ*B phosphorylation and degradation, which occurs in presence of NEMO-WT, were fully inhibited in NEMO-A323P just like in NEMO-KO cells (Supplementary Figure S1b). Nevertheless, all three cell lines were equally able to induce ERK and JNK phosphorylation upon TNF stimulation, demonstrating that the lack of TNF-induced NF-*κ*B activation in NEMO-A323P-expressing cells was not due to unrelated cell-intrinsic defects (Supplementary Figures S1c).

Remarkably, although both, NEMO-KO and cells expressing NEMO-A323P, are equally unable to activate NF-*κ*B, their susceptibility to cell death was significantly different, as NEMO-KO cells treated with TNF displayed an approximately threefold increase in the percentage of Annexin-V-stained cells, compared with NEMO-A323P cells that were only marginally sensitive to TNF-induced apoptotic cell death (Figure 1b). Interestingly, TNF-induced death of NEMO-KO cells was almost completely prevented by the pan-caspase inhibitor zVAD-fmk (zVAD), which efficiently blocked TNF-induced Caspase-8 activity in NEMO-KO cells (Figures 1c and d). In contrast, in NEMO-A323P cells a rapid and exacerbated cell death after treatment with TNF/zVAD was detected (Figure 1c). This cell death was blocked by the RIPK1 kinase inhibitor Nec-1 (Figure 1c).^29^ In line with a protective role of NEMO, NEMO-KO cells became more sensitive to cell death after 24 h of TNF/zVAD treatment compared with NEMO-WT (Supplementary Figure S1d). Nevertheless, A323P-NEMO-expressing cells that were almost completely dead after 6 h of TNF/zVAD treatment, after 24 h of stimulation became further sensitized to TNF/zVAD but showed no sensitization to TNF-induced cell death (Supplementary Figure S1d).

Collectively, these data show that despite the complete absence of NF-*κ*B activation, cells expressing NEMO-A323P were not sensitive to TNF-induced apoptosis but they exhibited a high sensitivity to cell death induced by TNF and zVAD.

### NEMO-A323P interferes with the caspase activation process

**Figure 2:**
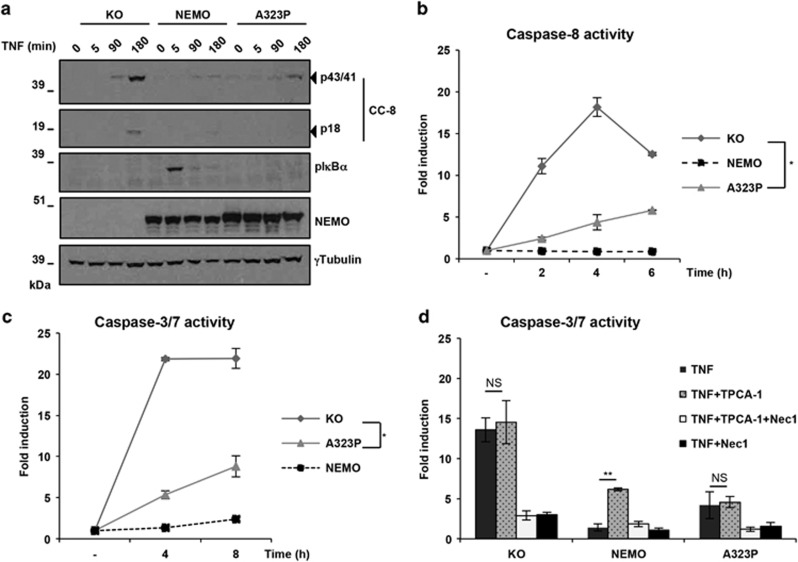
The NEMO-A323P interferes with the TNF-induced Caspases activation process in an IKK-independent manner. (**a**) KO, NEMO and NEMO-A323P cells were treated with 10 ng/ml TNF for the indicated times (min: minutes). Cells were lysed and subjected to immunoblot analysis for the indicated proteins. One representative of two independent experiments is shown. (**b** and **c**) KO, NEMO and NEMO-A323P cells were treated with TNF for the specified time periods (h: hours) to study the effect of TNF-induced Caspases activation. Time course of Caspase-8 and Caspase-3/7 proteolytic activity was calculated. In (**d**), the same cells were treated for 6 h with TNF alone or with TPCA-1. Caspase-3/7 proteolytic activity was calculated. Data are presented as mean±S.E.M. (three independent experiments performed). *P-value<0.01 (Student's *t*-test)

Our data showing that NEMO-KO cells are highly sensitive to TNF-induced apoptosis are in accord with previous findings.^10^ However, the fact that cells expressing NEMO-A323P were protected from TNF-induced death was unexpected. Since there seemed to be a decisive difference in caspase activity between these cells and NEMO-KO cells, we next investigated whether, and if so how, NEMO-A323P interferes with the TNF-mediated activation of the proteolytic cascade conveyed by caspases. As shown in Figure 2a, TNF treatment induced Caspase-8 cleavage (CC8) in NEMO-KO cells, yet the Caspase-8 cleavage was drastically reduced in NEMO-A323P-expressing cells (Figure 2a). Accordingly, we found that while TNF treatment of NEMO-KO cells resulted in activation of the Caspase-8 and the executioner Caspases 3 and 7, caspase activation was significantly reduced in NEMO-A323P-expressing cells (Figures 2b and c).

Although A323P-NEMO is able to bind to the IKK1/2 (Supplementary Figure S2a), the absence of NF-κB activity is caused by the inability of A323P-NEMO to recognize ubiquitin.^27^ Knowing that IKK-kinases prevent RIPK1 kinase-dependent apoptotic cell death, we next evaluated the effect of TPCA-1, an IKK inhibitor, on TNF-induced cell death in NEMO-KO, A323P-NEMO and NEMO-WT cells. As previously reported,^23^ pre-treatment with TPCA-1 sensitized NEMO-WT cells to RIPK1 kinase-dependent apoptosis (Figure 2d and Supplementary Figure S2b). In line with the absence of IKK-mediated NF-κB activity in both NEMO-KO and A323P-NEMO expressing cells, we found no additional effect on cell death and caspase activation after treatment with TNF in combination with the IKK inhibitor (Figure 2c and Supplementary Figure S2b).

Thus, the expression of the NEMO-A323P mutant, although unable to activate NF-*κ*B, prevents activation of Caspase-8 and, consequently, of downstream caspases upon TNF stimulation independently of the kinase activities of IKK1 and 2.

### NEMO-A323P inhibits RIPK3 recruitment to the pro-apoptotic FADD-containing complex

Our results so far showed that cells expressing NEMO-A323P are protected from apoptosis, yet surprisingly sensitive to necroptosis in the presence of zVAD, indicating that NEMO can act as a switch that determines cell fate during TNF/zVAD stimulation. In order to understand the mechanism by which this occurs, we analyzed the composition of the TNF-membrane-bound TNF-R1 complex I and the cytoplasmic complex II.

**Figure 3:**
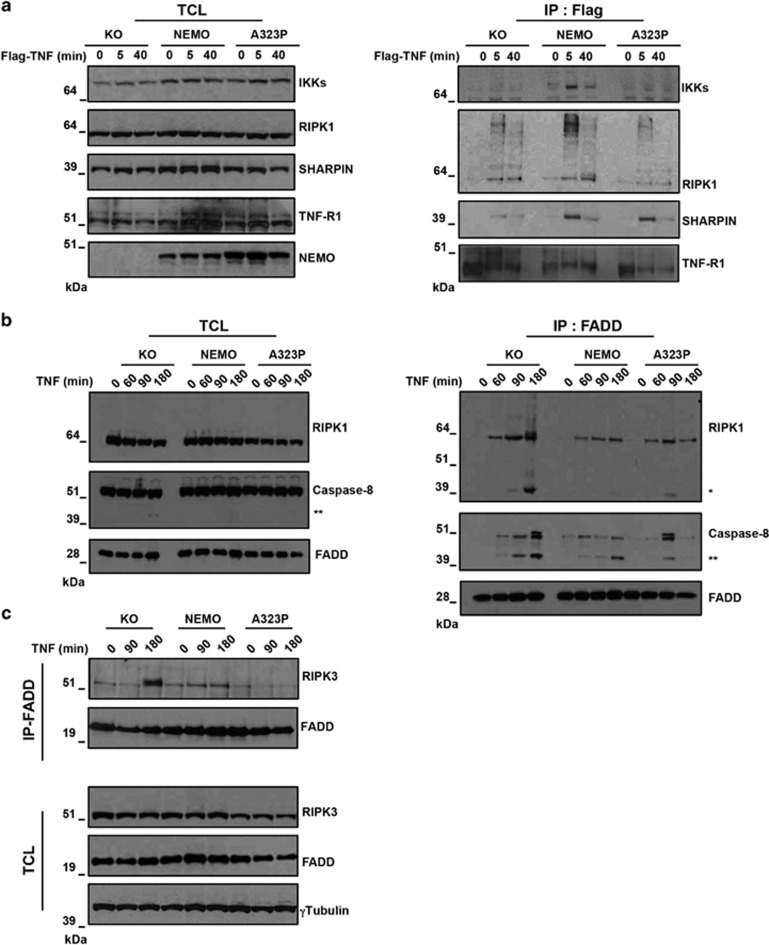
The presence of NEMO or NEMO-A323P prevents RIPK3 from integrating FADD-containing complex II. (**a**) Endogenous complex I pull-down was performed by Flag-TNF immunoprecipitation in KO, NEMO and A323P-NEMO cells upon different periods of Flag-TNF stimulation (min). Immunoblot analysis for the indicated proteins is shown. (**b** and **c**) KO, NEMO and NEMO-A323P cells were stimulated with TNF (10 ng/ml) for the indicated periods (min). Complex II was isolated by an FADD immunoprecipitation. Immunoblot analysis was performed to reveal the indicated proteins. Single asterisk indicates modification of RIPK1 when caspases are active and double asterisks indicate cleaved form of Caspase-8. A representative immunoblot revealing the levels of the indicated proteins is shown. IP, immunoprecipitation; TCL, total cell lysate

The analysis of the TNF-R1 complex I revealed that the IKK complex was recruited to TNF-R1 complex I only in cells expressing NEMO-WT but not in NEMO-KO neither in NEMO-A323P (Figure 3a). In contrast, a stimulus-dependent recruitment of RIPK1 and SHARPIN into complex I was evident in all cell types after 5 min of TNF exposure (Figure 3a and Supplementary Figure S3). Thus, NEMO-A323P is unable to recruit IKK but does not interfere with recruitment of the upstream factors RIPK1 and SHARPIN to the receptor, explaining the block in TNF-mediated NF-κB activation in these cells.

We next sought to establish whether the presence of NEMO-A323P affects the composition and functionality of the complex II formed upon TNF stimulation, promoting caspase activation. To this aim we immunoprecipitated the complex II component FADD and observed that 3 h after TNF stimulation, NEMO-KO cells exhibited substantially enhanced recruitment of Caspase-8 and RIPK1 to FADD-containing complex as compared with NEMO-WT (Figure 3b). Strikingly, recruitment of Caspase-8 and RIPK1 to complex II in NEMO-A323P-expressing cells was comparable to that observed in cells expressing NEMO-WT, indicating that NEMO modulates FADD-containing complex composition and that the A323P mutant retains this ability. Of note, an increased Caspase-8-dependent RIPK1 cleavage was revealed in NEMO-KO cells as compared with NEMO-A323P cells after TNF administration, further confirming an enhanced Caspase-8 activation in these cells (Figure 3b). The evidence that NEMO-KO cells are more prone to form FADD-containing complex upon TNF stimulation than NEMO-WT and NEMO-A323P cells was further evidenced by the fact that the recruitment of RIPK3 to FADD-containing complex after 3 h of stimulation was only evident in NEMO-KO cells (Figure 3c).

Together, these data show that NEMO, independently from its ubiquitin-binding domain, prevents recruitment of Caspase-8 and RIPK3 to the cell death-inducing complex II as revealed in NEMO-A323P cells.

### RIPK3 is able to phosphorylates MLKL outside the FADD-containing complex

**Figure 4:**
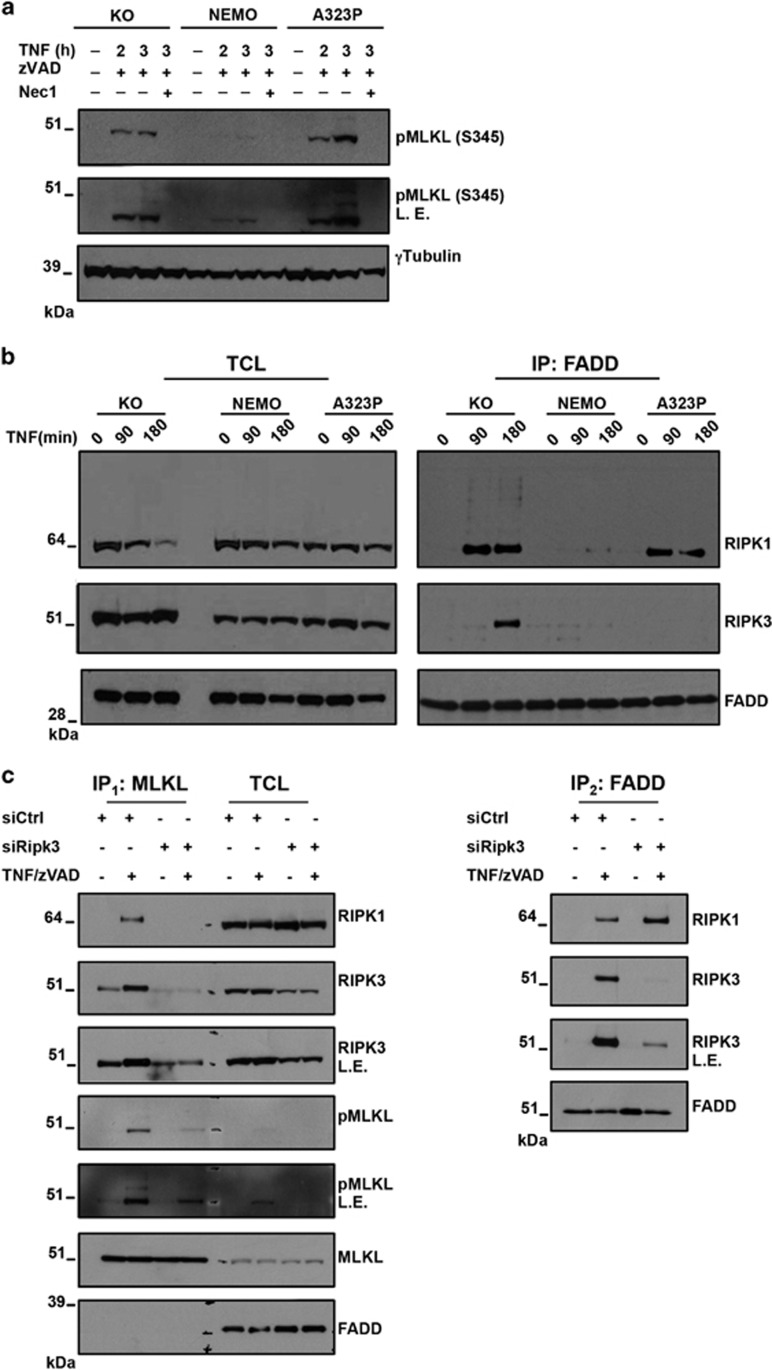
Ser-345 MLKL phopshorylation by RIPK3 is mediated by a RIPK1/RIPK3/MLKL complex. (**a**) KO, NEMO and NEMO-A323P cells were pre-incubated 1 h with the indicated compounds followed by TNF stimulation for the indicated time period (hour: h). Immunoblot analysis for the indicated proteins is shown. L.E., long exposure. (**b**) KO, NEMO and NEMO-A323P cells were pre-treated 1 h with zVAD followed by a time course (min) with 10 ng/ml TNF. Complex II was isolated by an FADD immunoprecipitation. Immunoblot analysis was performed to reveal the indicated proteins. (**c**) siCTR and siRIPK3 Nemo^(−)^ MEF cells were treated with TNF/zVAD as indicated. The MLKL complex was isolated by a first immunoprecipitation against MLKL (IP_1_). The supernatants derived from IP_1_ were subjected to a second IP against FADD (IP_2_) to isolate the complex II. Ifmmunoblot analysis was performed to reveal the indicated proteins

In the absence of caspase activity, death receptors can still mediate cell death via programmed necrosis.^30, 31^ The fact that NEMO-A323P-expressing cells did not die after TNF treatment, but were highly sensitive to necroptotic cell death upon stimulation with TNF in the presence of zVAD, demonstrated that (i) the residual activity of Caspase-8 in NEMO-A323P cells was sufficient to prevent necroptosis following TNF stimulation and (ii) that inhibition of this residual Caspase-8 activity by zVAD promoted massive necroptotic cell death. It was previously shown that necrosome activation requires formation of the RIPK1–RIPK3 heterodimeric amyloid scaffold, leading to activation of RIPK3 which in turn induces necroptosis through MLKL phosphorylation at Ser-345.^16, 17, 32^ Consistent with our findings, the NEMO-A323P cells exhibited significantly enhanced phosphorylation of MLKL after 3 h of stimulation with TNF/zVAD as compared with NEMO-KO cells (Figure 4a). The simultaneous inhibition of RIPK1 kinase activity by Nec-1 completely inhibited MLKL phosphorylation (Figure 4a) and also blocked cell death (Figure 1c).

The original proposed model of necroptosis induction, encompasses the stabilization of RIPK1/FADD/Caspase-8, the subsequent RIPK3 recruitment to the inactive FADD-containing complex which results in activation of MLKL and execution of necroptosis.^33^ Nonetheless, a new scenario where a RIPK1/RIPK3/MLKL complex is formed in parallel to the FADD-containing complex has been proposed.^16, 34^ To gain mechanistic insight on how the phosphorylation of MLKL occurs, we investigated whether RIPK3 is recruited to the FADD complex in NEMO-A323P cells after TNF/zVAD stimulation. Upon TNF stimulation, RIPK1 co-immunoprecipitated with FADD in both NEMO-KO and NEMO-A323P, but not in NEMO-WT-expressing cells (Figure 4b). Surprisingly, however, the recruitment of RIPK3 was easily detectable only in NEMO-KO cells (Figure 4b). In addition, we did not find MLKL in the FADD-containing complex (Supplementary Figure S4).

The evidence that NEMO-A323P cells were significantly sensitized to necroptosis but did not show recruitment of RIPK3 to the FADD-containing complex prompted us to investigate the possibility that the active necrosome is a RIPK1/RIPK3/MLKL complex that forms in parallel with but independently of the FADD-containing complex. To determine whether the recruitment of RIPK3 to the FADD-containing complex that we found only in NEMO-KO cells was actively inducing necroptosis, we assessed the formation of both the MLKL/RIPK3/RIPK1 necroptosis-inducing signaling complex and the FADD/Caspase-8 apoptosis inducing complex in NEMO-KO by performing sequential co-immunoprecipitation assays. The first MLKL immunoprecipitation showed that the MLKL/RIPK3 complex was present in resting cells but the amount of RIPK3 that was associated with MLKL increased upon necroptosis induction and was also associated with RIPK1 recruitment. Importantly, these events were linked to MLKL phosphorylation and were blocked after RIPK3 knockdown. In contrast, FADD is not recruited to the RIPK3/MLKL complex (Figure 4c). The second FADD immunoprecipitation showed that RIPK3 and RIPK1 were associated with FADD-containing complex but this complex is not the one in which the phosphorylation of MLKL occurred.

Together with the higher MLKL phosphorylation seen in NEMO-A323P-expressing cells (Figure 4a), these data show that RIPK3 and MLKL recruitment to the FADD-containing complex is not required for RIPK3 activation and induction of necroptosis but, instead, an independent RIPK1/RIPK3/MLKL is actively inducing necroptosis.

### In the absence of NEMO, RIPK3 promotes apoptosis

**Figure 5:**
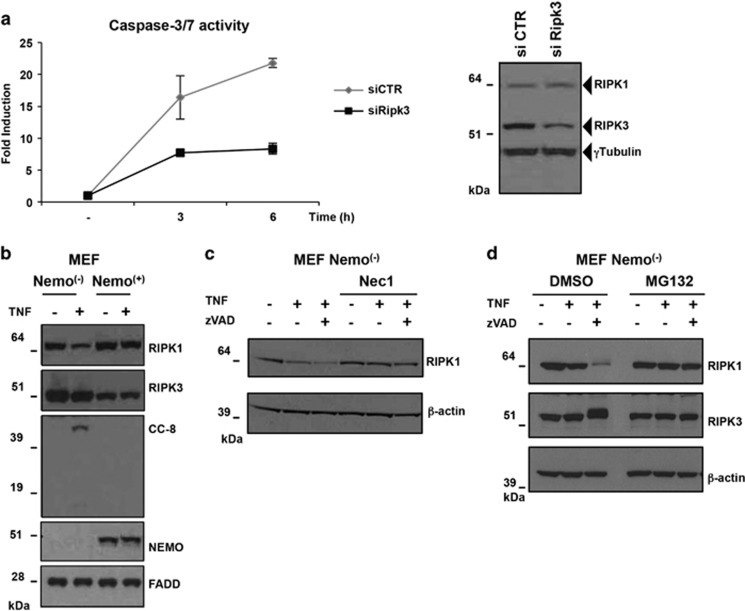
RIPK3 has a positive effect on the activation of Caspases in the absence of NEMO. (**a**) siCTR and siRIPK3 Nemo^(−)^ MEF cells were treated with TNF (10 ng/ml) and Caspase-3/7 activation was measured in function of time (h). Error bars represent S.D. of quadruplicates of one representative experiment. Immunoblot analysis was performed to determine the RIPK3 protein level. (**b**) MEFs Nemo^(−)^ or Nemo^(+)^ were stimulated with TNF (10 ng/ml) for 3 h. Immunoblot analysis was performed to determine the indicated protein levels. (**c** and **d**) MEFs Nemo^(−)^ cells were pre-treated with Nec1 for 1 h and then stimulated with TNF (10 ng/ml) alone or in combination with zVAD, for the indicated time. Immunoblot analysis was performed to determine the indicated protein levels

Having demonstrated that RIPK3 recruitment to the FADD-containing complex only occurred in NEMO-KO cells and that this event is not necessary for induction of MLKL phosphorylation, we decided to investigate the functional significance of this event. Sometimes overlooked, RIPK3 was initially described as a pro-apoptotic molecule as its ectopic expression in mammalian cells caused apoptosis.^35, 36^ Recently, it was reported that absence of RIPK3 reduced Caspase-8 activity when cIAP1/2 were inhibited by SMAC mimetics.^37, 38^ Furthermore, the characterization of RIPK3 inhibitors and of the kinase-inactive RIPK3 mutant (D161N) revealed a surprising conformational change that promotes a different form of RHIM-mediated interaction between RIPK1 and RIPK3 leading to FADD/Caspase-8 binding and apoptosis.^39, 40^ We therefore suspected that RIPK3 recruitment to the FADD complex in NEMO-KO cells might be responsible for enhanced TNF-induced caspase activation (Figures 2b and c). Accordingly, the knockdown of Ripk3 by siRNA resulted in decreased Caspase-3 and -7 activity in NEMO-deficient MEF cells following TNF stimulation (Figure 5a), indicating that RIPK3 can promote activation of apoptosis, independently of its role in promoting necroptosis.

Interestingly, upon TNF stimulation the protein level of RIPK1 in the absence of NEMO was affected (Figure 5b), suggesting that NEMO may be involved in the regulation of RIPK1 stability. This observation is in accordance with previous work showing that, in the absence of NEMO, RIPK1 is degraded following TNF treatment.^4^ Despite the fact that TNF-induced Caspase-8 activation results in RIPK1 cleavage and its inactivation (Figure 3b), when Caspase-8 was inhibited by zVAD a dramatic reduction in RIPK1 level in the total extracts of NEMO-deficient cells (Nemo^(−)^) was observed (Figure 5c). Notably, pre-treatment of these cells with either Nec1, or the proteasome inhibitor MG132, prevented the TNF-induced decrease in RIPK1 levels in NEMO-deficient cells (Figures 5c and d). Together, these data indicate that the absence of NEMO sensitizes to apoptosis in a RIPK3-dependent manner and causes a partial protection from necroptosis induced by a complete destabilization of RIPK1 after stimulation with TNF/zVAD.

## Discussion

NEMO is the regulatory subunit of the IKK complex, essential for canonical TNF-dependent NF-*κ*B activation. NEMO-deficient cells preferentially undergo apoptosis and caspase inhibition is required to undergo RIPK1 kinase-dependent programmed necrosis. It was previously proposed that the activation of NF-*κ*B is a ‘late' pro-survival checkpoint in the TNF-R1 pathway and that NEMO also has an ‘early' pro-survival function consisting in restraining the death-inducing activity of RIPK1 upon TNF stimulation, in a ubiquitin-dependent fashion.^10^ In the absence of NEMO, RIPK1 engages via FADD with Caspase-8 leading to apoptosis or slow promoting necroptosis when caspase activity is blocked.^10, 41^ Nevertheless, a scaffolding role of NEMO in the formation of the pro-necroptotic TNF-induced complex in MEFs has also been proposed.^22^ So far it has been difficult to conciliate the atypical roles of NEMO in suppressing and activating cell death, considering its prominent role as a non-catalytic subunit of the IKK complex, regulating the signal-dependent NF-*κ*B activation and the IKK-mediating antiapoptotic effects.^23^

We have examined how the NUB domain mutant, NEMO-A323P, influences TNF-induced signaling. Although NEMO-A323P and NEMO-KO cells exhibit a similar defect in NF-*κ*B activation, as both cell lines are unable to recruit IKK to complex I of TNF-R1 signaling, we observed a dramatic difference between cells lacking NEMO or expressing NEMO-A323P in terms of sensitivity to TNF-induced death. In contrast to NEMO-KO cells that are prone to TNF-induced apoptosis, the presence of NEMO-A323P induces: (i) a strong but not complete protection from TNF-induced apoptosis, independent from IKK kinase activity, and (ii) a profound sensitization to necroptosis when Caspase-8 is inhibited.

Our experiments demonstrate that the composition of the cytosolic FADD-containing complex in NEMO-KO and NEMO-A323P cells differs in its ability to promote caspase-dependent apoptosis. For the first time we have shown that recruitment of RIPK3 to the FADD-containing complex is detectable only in the absence of NEMO. We propose that this event enhances the pro-apoptotic response by bolstering caspase activation. In contrast, the presence of the NUB mutant NEMO-A323P is sufficient to inhibit RIPK3 recruitment to the pro-apoptotic complex, resulting in partial protection from apoptosis following TNF treatment. Interestingly, it has recently been reported that RIPK3, independently from its kinase activity, is able to contribute to RIPK1-dependent apoptosis induced by treatment with TNF and SMAC mimetics.^38^

Having demonstrated here that RIPK3 may positively regulate RIPK1-dependent apoptosis in the absence of NEMO, it remains to be clarified whether the presence of NEMO or NEMO-A323P inhibits RIPK3 pro-apoptotic activity through a direct binding or, indirectly, perhaps via the interaction of NEMO with RIPK1. Notably, the absence of NEMO induces a global destabilization of RIPK1 after TNF treatment. This effect is Caspase-8-independent as it is not corrected by treatment with zVAD. However, we have found that the expression of NEMO-A323P prevents RIPK1 degradation. As the RIPK1 scaffolding role and kinase activity are both important in preventing apoptosis in NEMO-deficient hepatocytes,^42^ we speculate that the ubiquitin-binding mutant NEMO-A323P is unable to bind to ubiquitinated RIPK1 at complex I but that it can still interact with RIPK1 via its pseudo-*α*-helix.^4, 25, 27^ This interaction could in turn enhance the RIPK3 kinase activity and also explain the exacerbated cell death we observed in NEMO-A323P cells, when an inhibition of Caspase-8 activity occurs.

The inhibition of Caspase-8 promotes TNF-induced necroptosis by preserving the integrity of RIPK1 and RIPK3. However, the precise mechanism of RIPK3-mediated phosphorylation of MLKL is still unclear.^43^ Our data reveal that the phosphorylation of MLKL is barely detectable in NEMO-KO cells after TNF/zVAD stimulation. We have observed a strong association between RIPK3 and FADD in NEMO-deficient cells but we have not detected MLKL in the FADD-containing complex of NEMO-KO cells, indicating that RIPK3 recruited to this complex is not associated with MLKL. This has also been suggested by the fact that RIPK3 recruitment to the FADD-containing complex also occurs after TNF treatment, a condition in which RIPK3/MLKL-mediated cell death is inhibited. Instead, MLKL phosphorylation happens faster and is strongly amplified in NEMO-A323P cells upon TNF/zVAD stimulation leading to rapid cell death. In these cells RIPK3 is not recruited to the FADD-containing complex and there is no change in the RIPK1 protein level upon stimulation. Importantly, in the NEMO-KO cells, we have shown that the RIPK1/RIPK3/MLKL complex forms independently of the FADD-containing complex and the death signal progresses after the recruitment of additional molecules of RIPK3 that ultimately induces MLKL phosphorylation.

**Figure 6:**
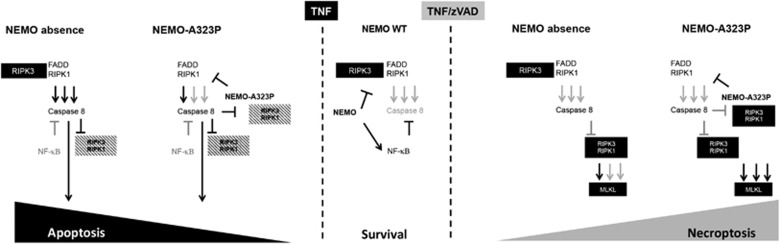
Proposed model of the balance between apoptosis (left panel), survival (central panel) and necroptosis (right panel), in the absence of NEMO, in the presence of NEMO-WT and of the NEMO-A323P, respectively

Collectively, our results demonstrate that the absence of NEMO is a condition that is different from the presence of mutated form of NEMO that is incapable of binding ubiquitin in terms of sensitivity to different kinds of cell death (Figure 6). In the presence of NEMO-WT upon TNF or TNF/zVAD treatment, cell death is inhibited by the consequence of NF-*κ*B activation and by IKK-mediated inhibition of RIPK1's kinase activity (Figure 6, central panel). In the absence of NEMO, TNF treatment induces assembly of the RIPK1/FADD/Caspase-8 complex that includes RIPK3. Despite the presence of RIPK3, this complex only induces apoptosis. The presence of NEMO-A323P prevents RIPK3 recruitment to this RIPK1/FADD/Caspase-8 complex with the result that its apoptosis-inducing capacity is limited. This latter condition is similar to the silencing of RIPK3 by siRNA in NEMO-deficient cells (Figure 6, left panel). However, in the absence of Caspase-8 activity, the presence of NEMO-A323P promotes RIPK3-mediated MLKL phosphorylation most probably by preventing the degradation of RIPK1 (Figure 6, right panel).

Although the role of necroptosis in IP remains unclear, it is possible that the presence of mutated NEMO inducing a major sensitivity to necroptosis triggers some severe aspects of IP, such as brain atrophy, brain edema and retinal abnormalities. Furthermore, analyzing to what extent RIP kinases may be directly involved, and whether necroptosis might contribute to the pathogenesis of IP, is an intriguing question that deserve to be addressed in future research.

## Materials and Methods

### Retroviral transduction of cells

Coding sequence-verified PCR fragments of NEMO and NEMO-A323P were inserted into the pBABE vector containing the porumycin resistance as a selectable marker. The pBABE-NEMO vectors and empty pBABE-puro were used to transfect LinX packaging cells, using a standard CaPO_3_ procedure. Conditioned media collected from the transfected LinX cells after 48 h were filtered through a 0.45-mm pore size syringe filter. This mixture, supplemented with polybrene (8 mg/ml; Sigma-Aldrich, St. Louis, MO, USA), was added to recipient cells, NEMO^(−)^ MEF^44^ that had been plated at 5 × 10^5^ cells per 100  mm dish the day before infection. Cells were cultured with viral supernatants for 12 h. After 12 h, the cells were subjected to selection in 2 mg/ml puromycin for 7–14 days. Clones of puromycin-resistant cells were obtained and analyzed for NEMO expression by real-time and immunoblot assays. Nemo-KO (pBABE-empty), NEMO-WT and NEMO-A323P reconstituted MEFs were cultured in Dulbecco's modified Eagle's medium (Invitrogen, Carlsbad, CA, USA) supplemented with 10% fetal bovine serum, penicillin (50 U/ml), streptomycin (50 mg/ml) and puromycin (2 mg/ml).

### Cell survival and Caspase assay

Cell survival analysis was performed using the Cell Titer-Glo Luminescent Cell Viability Assay kit (Promega, Madison, WI, USA) following the manufacturer's instruction with minor modification.

Caspase-8 or Caspase-3/7 activity was measured using the Caspase-Glo 8 or Caspase-Glo 3/7 assay kits (Promega) that uses a luminogenic caspase-8 (LETD) or caspase-3/7 (DEVD) substrate. In both assays, MEF cells were seeded onto 96-well plates at a density of 8 × 10^3^ cells/well, in triplicates. Twenty-four hours later, cells were pre-treated with zVAD (20 *μ*M) (Sigma), Necrostatin-1 (30 *μ*M) (Sigma), TPCA-1 (5 *μ*M) (Tocris, Minneapolis, MN, USA) or DMSO (Sigma) for 1 h and then stimulated with TNF (20 ng/ml) for period indicated in the figures. Luminescent reading was carried on the 96-well plate reader (GloMax 96 Microplate Luminometer; Promega). Apoptotic cells, after TNF tretment, were identified by double staining with the Annexin V FITC kit, according to the manufacturer's instructions (Miltenyi Biotec, Bergisch Gladbach, Germany).

### Cell stimulation and immunoprecipitation

For the FADD-containing complex (FADD-IP), 3 × 10^6^ MEF cells were seeded the day before in a 150 mm diameter culture dish. Cells were stimulated with recombinant TNF (10 ng/ml) for the indicated times. Where indicated, before TNF stimulation, cells were pre-treated with zVAD (Sigma (20 mM)) for 30 min. Cells were lysed in IP-lysis buffer (30 mM Tris-HCl, pH 7.4, 120 mM NaCl, 2 mM EDTA, 2 mM KCl, 10% glycerol, 1% Triton X-100, EDTA free protease-inhibitor cocktail (Roche, Indianapolis, IN, USA) and 1 × phosphatase inhibitor (Sigma)) at 4 °C for 30 min. After incubation on ice and centrifugation at 20 000 r.c.f. for 10 min, the supernatant was recovered and the protein concentration determined. Proteins were either directly analyzed, after electrophoresis, by western blotting or immunoprecipitated. Complex II isolation was performed by using anti-FADD antibody (Santa Cruz Biotechnologies, Dallas, TX, USA) coupling to protein G-Agarose beads (Sigma) at 4 °C under rotation. The following day, the sepharose beads were washed three times with IP-lysis buffer. After the final wash, proteins were eluted by boiling in reducing sample buffer and analyzed by western blotting. For the MLKL-IP the anti-MLKL (Abgent, San Diego, CA, USA) was coupled to protein A-agarose beads. For complex I isolation, cells stimulation performed with 1 *μ*g/ml Flag-TNF in serum-free medium for the indicated times. Cells were lysed in IP-lysis buffer (30 mM Tris-HCl, pH 7.4, 120 mM NaCl, 2 mM EDTA, 2 mM KCl, 10% glycerol, 1% Triton X-100 and EDTA free protease-inhibitor cocktail (Roche)) at 4 °C for 1 h. Flag-TNF (1 *μ*g) was added to the lysates of non-stimulated control samples. Subsequently, the lysates were centrifuged at 19 000 r.c.f. for 20 min and the TNF-RSC was immunoprecipitated using M2 beads (Sigma) overnight at 4 °C. The following day, the beads were washed three times with 1 ml IP-lysis buffer. Proteins were eluted by boiling in reducing sample buffer and analyzed by immunoblotting. Hek293 NEMO-null cells were transfected as previously described.^26^ Membranes were probed with primary antibodies against following proteins: NEMO (Santa Cruz; sc-8330), IKK-*α*/*β* (Santa Cruz; sc-7607), RIPK1 (BD Biosciences, San Jose, CA, USA; 610459), Caspase-8 (Enzo LifeSciences), RIPK3 (Enzo LifeSciences), Phospho-JNK (Cell Signaling Technology, Denver, MA, USA; 9251), Phospho-ERK (Cell Signaling; 9101), cleaved Caspase-8 (Cell Signaling; 4927), *γ*-tubulin (Sigma-Aldrich), phospho-I*κ*B*α* (Cell Signaling; 7246), MLKL and Phospho-MLKL(S345) (Abcam, Cambridge, MA, USA; 194699 and 196436), TNF-R1 (Abcam; 19139), (M2) FLAG (Sigma; F3165). Membranes were then incubated with secondary HRP-coupled antiboidies (Bio-Rad Laboratories, Hercules, CA, USA).

### RNAi-mediated knockdown

MEF cells were seeded at 2 × 10^5^/cells well in six-well plates. After 6 h of incubation at 37 °C, the cells were transfected with 5 nM siRNA-targeting RIPK3 or 5 nM non-targeting SiRNA negative control (Silencer select; Ambion, Life technologies, Carlsbad, CA, USA) by using Lipofectamine RNAiMAX (Invitrogen), according to the manufacturer's instructions. After 24 h, the cells were stimulated with TNF and caspase activity was measured as described above. Knockdown efficiency was tested by immunoblotting.

### Quantitative RT-PCR

MEF cells were left untreated or treated with 10 ng/ml TNF for the indicated times. Total RNA was extracted from the MEFs using the RNeasy Mini Kit (Qiagen, Venlo, Netherlands) according to the manufacturer's instructions with on-column DNase digestion Kit. Total RNA was used to make cDNA using Superscript III First Strand Synthesis System for RT-PCR (Invitrogen). Steady-state mRNA abundance was determined by real-time PCR by using Power SYBR Green PCR Master Mix (Applied Biosystems, Waltham, MA, USA) on the 7900HT Fast Real Time PCR System (Applied Biosystems, Foster City, CA, USA), as described elsewhere,^45^ using the following primers: Icam1 (5′-TTCACACTGAATGCCAGCTC-3′; 3′-GTCTGCTGAGACCCCTCTTG-5′); Nfkbia (5′-CTGCAGGCCACCAACTACAA-3′ 3′-CAGCACCCAAAGTCACCAAGT-5′); Hprt (5′-GGCTTACCTCACTGCTTTCC-3′ 5′-CTGGTTCATCATCGCTAATCAC-3′).

## Abbreviations

A: alanin
CC8: cleaved caspase-8
cIAP: cellular inhibitor of apoptosis protein
DD: death domain
FADD: Fas-associated death domain-containing protein
FLICE: FADD-like interleukin-1 *β*-converting enzyme
ICAM: intercellular adhesion molecule
I*κ*B: inhibitor of nuclear factor-*κ*B
IKK: inhibitor of -*κ*B kinase
IKKs: IKK1 and 2
IP: incontinentia Pigmenti
JNK: jun N-terminal kinase
KO: knockout
LUBAC: linear ubiquitin chain assembly complex
MAPK: mitogen-activated protein kinase
MEFs: mouse embryonic fibroblasts
MLKL: mixed lineage kinase domain-like protein
NEMO: NF-*κ*B essential modulator
Nec1: Necrostatin-1
NF-*κ*B: nuclear factor-*κ*B
NUB: NEMO ubiquitin binding
P: Prolin
RHIM: RIP homotypic interaction motifs (RHIMs)
RIPK: receptor interacting protein kinase
Ser: serin
SiRNA: small interfering RNA
SMAC: second mitochondrion-derived activator of caspases
TAB1/2: TAK1 binding protein 1/2
TAK1: TGF—activated kinase 1
TNFR: tumor necrosis factor receptor
UBAN: ubiquitin-binding domains found in ABINs and NEMO
zVAD: z-Val-Ala-DL-Asp-fluoromethylketone
