# Supplementary figures and images for: NEMO regulates a cell death switch in TNF signaling by inhibiting recruitment of RIPK3 to the cell death-inducing complex II
Source: Cell Death Dis. 2016 Aug 25;7(8):e2346–. doi: 10.1038/cddis.2016.245 (PMC5108330; doi:10.1038/cddis.2016.245)

# Supplemental Figure 1

**a**

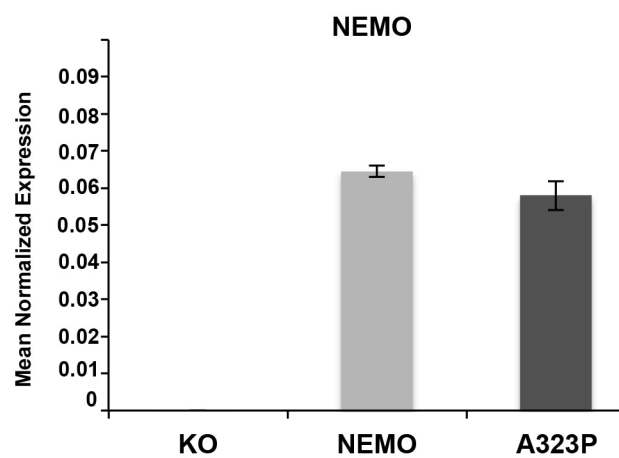

**b**

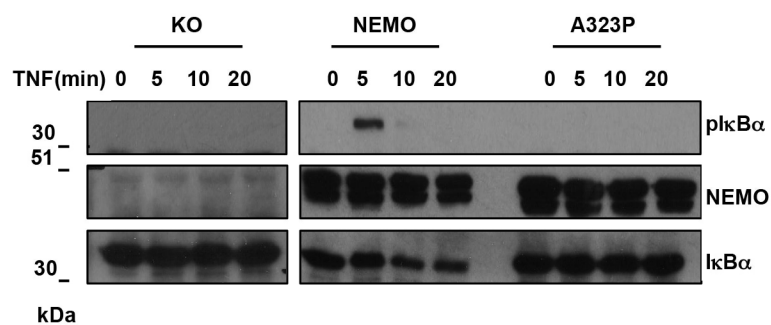

**c**

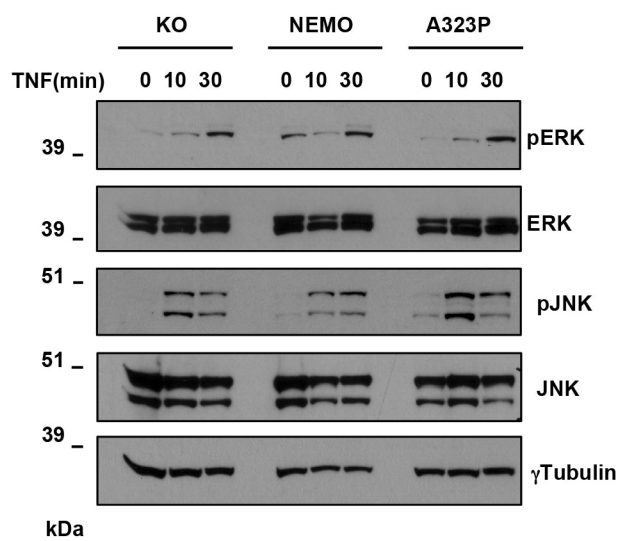

**d**

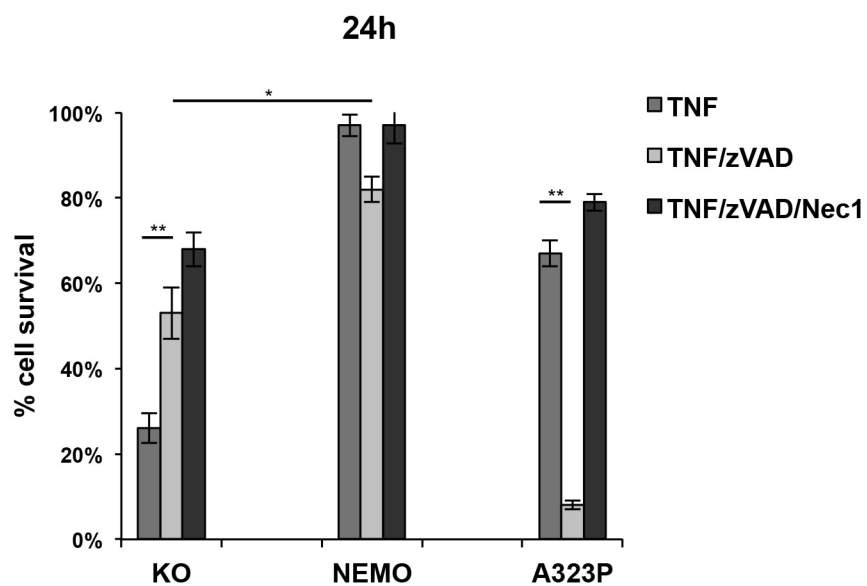

Supplement: Supplementary Figure 1 [file cddis2016245x1.pdf]

# Supplemental Figure 2

**a**

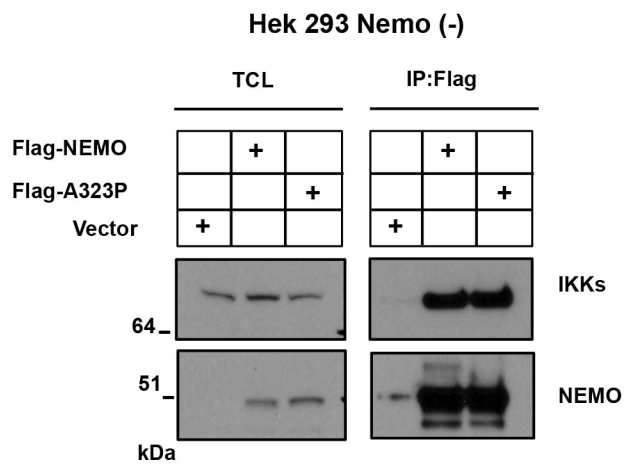

**b**

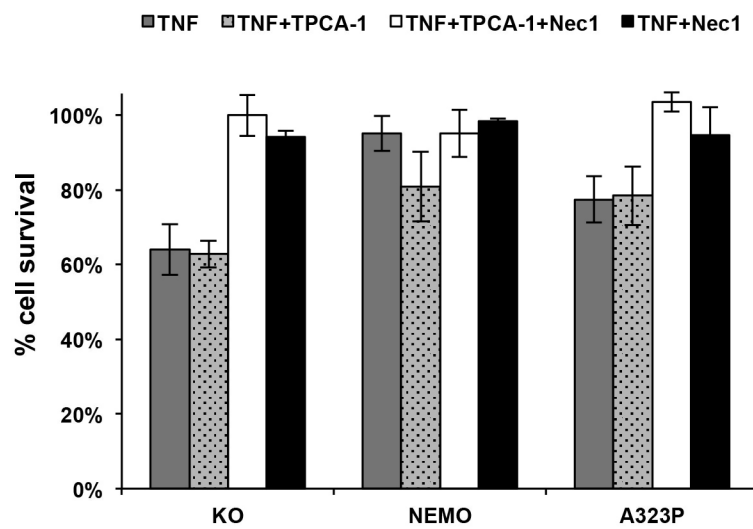

Supplement: Supplementary Figure 2 [file cddis2016245x2.pdf]

# Supplemental Figure 3

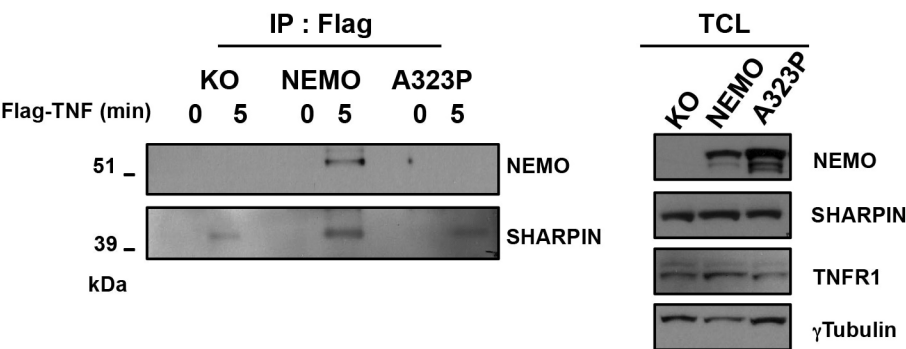

Supplement: Supplementary Figure 3 [file cddis2016245x3.pdf]

# Supplemental Figure 4

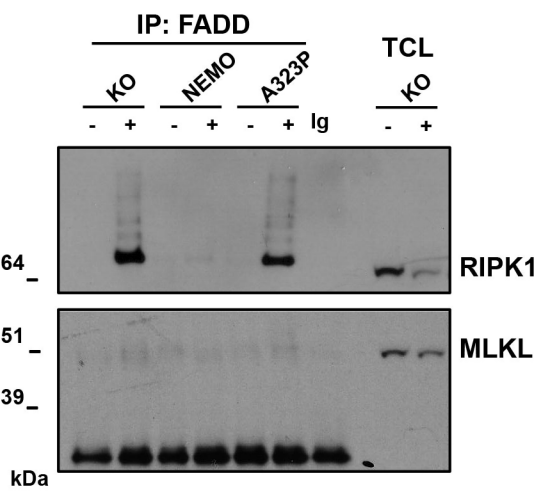

Supplement: Supplementary Figure 4 [file cddis2016245x4.pdf]
