# Supplementary material for: NEMO regulates a cell death switch in TNF signaling by inhibiting recruitment of RIPK3 to the cell death-inducing complex II
Source: Cell Death Dis. 2016 Aug 25;7(8):e2346–. doi: 10.1038/cddis.2016.245 (PMC5108330; doi:10.1038/cddis.2016.245)
Supplement: Supplementary Figure Legends [file cddis2016245x5.doc]

**Figure S1**

**TNF treatment induced MAPK and JNK phosphorylation in the NF-κB unresponsive KO and NEMO-A323P cells. (a)** Quantitative real-time PCR was performed in triplicate, and the relative abundance of NEMO transcript was calculated with respect to the expression of ribosomal protein 18s transcript. Error bars represent SD of triplicates of a triplicate experiment.  **(b, c)** KO, NEMO and NEMO-A323P cells were treated with 10 ng/ml of TNF at the indicated time points (min). Immunoblot analysis was performed to identify the level of the indicated proteins. **(d)** KO, NEMO and A323P expressing cells were preincubated for 1 hour with either DMSO, zVAD (20mM) or zVAD in combination with Nec1 [30µM], followed by 24 hours of TNF stimulation. Relative cell viability was assessed by determining ATP levels with CellTiter-Glo® after 24 hours of TNF treatment, as a percentage of untreated cells. Data are presented as mean ± SEM (three independent experiments performed), * p value < 0,01 **p value < 0,001 (Student’s t-test).

**Figure S2:**

**NEMO-A323P protects cells from TNF-induced cell death independently of IKK kinase activity.**

**(a)** Hek 293 NEMO-null cells (Hek 293 Nemo(-)) were tranfected with Flag-NEMO or Flag-A323P. NEMO was immunoprecipitated via Flag and the interaction with endogenous IKK1/2 (IKKs) was assessed by immunoblot. **(b)** KO, NEMO and NEMO-A323P cells were treated with TNF alone or in combination with TPCA-1. Relative cell viability was assessed by determining ATP levels with CellTiter-Glo® after 6 hours of TNF treatment, as a percentage of untreated cells.

**Figure S3**

**NEMO recruitment to the complex I**

Endogenous complex-I pull-down was performed by Flag-TNF immunoprecipitation in KO, NEMO and A323P-NEMO cells upon different periods of Flag-TNF stimulation (min). Immunoblot analysis for the indicated proteins is shown.

**Figure S4**

**MLKL is not recruited to the FADD-containing complex**

Immunoblot of the indicated components from anti-FADD immunoprecipitation of KO, NEMO and NEMO-A323P cells pretreated 1 hour with zVAD followed by 3h of TNF treatment.
